# Brain death and marginal grafts in liver transplantation

**DOI:** 10.1038/cddis.2015.147

**Published:** 2015-06-04

**Authors:** M B Jiménez-Castro, J Gracia-Sancho, C Peralta

**Affiliations:** 1 Centro de Investigación Biomédica en Red de Enfermedades Hepáticas y Digestivas, Institut d'Investigacions Biomèdiques August Pi i Sunyer IDIBAPS, Esther Koplowitz Center, Barcelona, Spain; 2 Barcelona Hepatic Hemodynamic Laboratory, IDIBAPS, CIBEREHD, Barcelona, Spain

## Abstract

It is well known that most organs for transplantation are currently procured from brain-dead donors; however, the presence of brain death is an important risk factor in liver transplantation. In addition, one of the mechanisms to avoid the shortage of liver grafts for transplant is the use of marginal livers, which may show higher risk of primary non-function or initial poor function. To our knowledge, very few reviews have focused in the field of liver transplantation using brain-dead donors; moreover, reviews that focused on both brain death and marginal grafts in liver transplantation, both being key risk factors in clinical practice, have not been published elsewhere. The present review aims to describe the recent findings and the state-of-the-art knowledge regarding the pathophysiological changes occurring during brain death, their effects on marginal liver grafts and summarize the more controversial topics of this pathology. We also review the therapeutic strategies designed to date to reduce the detrimental effects of brain death in both marginal and optimal livers, attempting to explain why such strategies have not solved the clinical problem of liver transplantation.

## Facts

- Brain death (BD) and marginal grafts are both key risk factors in clinical liver transplantation (LT).
- There is a significant disparity in results regarding the changes induced by BD as well as the use of marginal livers, and their relevance in LT.
- The strategies applied in LT have been mainly focused in the treatment with different hormones to stabilize the hemodynamic disorders associated with BD, whereas the strategies focused at protecting liver grafts are performed in non-BD surgical conditions.

## Open Questions

- Future prospective, randomized clinical studies and studies that include a sufficient number of marginal donors will be required to elucidate the effects of BD on marginal liver grafts, and to select the most appropriate marginal organs for transplant.
- Future research in experimental models of LT using BD donors is strongly required to understand the pathophysiology of BD, and, therefore, elucidate the consequences of BD.
- The experimental conditions focused not only on liver graft damage associated with transplantation but also on brain-dead donor, and should maximally mimic the clinical situation of LT to ultimately develop effective therapeutic strategies in this setting.

## Deceased Donation

**Figure 1:**
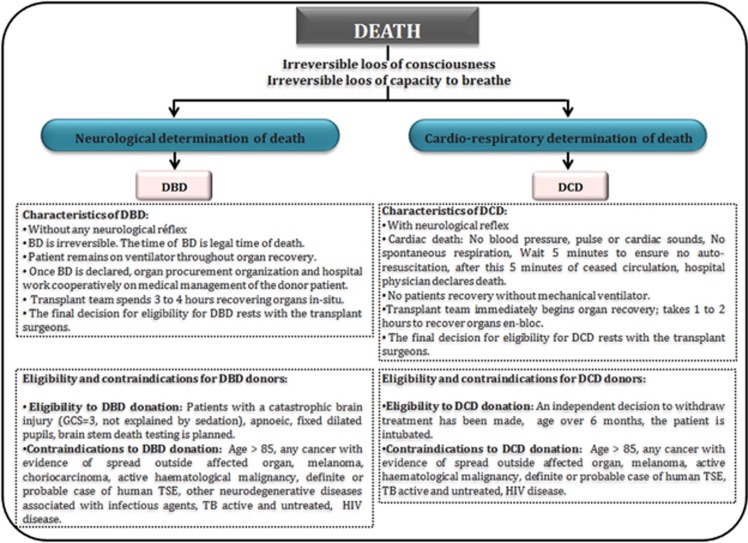
Comparison of DBDs and DCDs. Characteristics, eligibility and contraindications from DBD and DCD donors. GCS, Glasgow Coma Scale; HIV, human immunodeficiency virus; TB, tuberculosis; TSE, transmissible spongiform encephalopathy

Deceased donation includes two types of donation: donation after circulatory death (DCD) and donation after brain death (DBD). The fundamental distinction between DCD and DBD is the diagnosis of death. DCD describes the retrieval of organs for the purpose of transplantation, which follows death confirmed using circulatory criteria, and contrasts with the standard model for deceased donation, namely donation after the confirmation of death using neurological criteria^1, 2^ (Figure 1). The potential contribution of DCD to overall deceased donor numbers varies internationally and comprises between 4 and 20% of transplanted grafts among centers with high rates of use.^3^

BD has been defined as the irreversible loss of brain and brain stem function, usually caused by major hemorrhage, hypoxia or metabolic dysregulation.^4^ The diagnosis is based on a comprehensive neurologic assessment with the absence of brain stem reflexes and apnea. Electroencephalography, transcranial Duplex-ultrasound or cerebral angiography is required in cases of clinical examination uncertainty.^5^ The DBD is always ventilated before death and the heart remains beating at the time of retrieval, thus virtually eliminating any warm ischemic injury to donor organs.

## Pathophysiological Changes Occurring During and After BD

**Figure 2:**
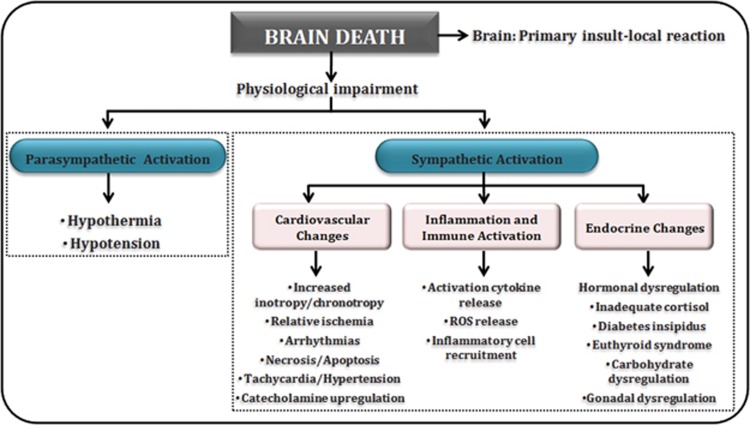
Pathophysiological changes occurring during and after BD. It begins with physiological impairment and consequently an alteration in sympathetic and parasympathetic branches. In the first, cardiovascular and endocrine changes and inflammation and immune activation are the most representative changes

BD is the terminal phase of a sequence of events frequently commencing with cerebral trauma or cerebrovascular hemorrhage. When the patient is declared brain dead, this chain of events has already affected all potential donor organs. Systemic and hormonal changes arise immediately when intracranial pressure (ICP) increases. Hemodynamic events, hormonal changes and inflammation and immune activation occur consequently to BD.^1, 6^ Figure 2 summarizes some of the pathophysiological events occurring during and after BD. However, it should be considered that there is a range of different results with regard to the changes induced by BD and their relevance in LT. Such differences may explain why the detrimental effects of BD in LT remain as an unresolved problem in the clinical practice.

It has been reported that in clinical situations, as a result of BD, massive catecholamine is systemically released, which causes an increase in heart rate and leads to vasoconstriction with increments in vascular resistance and blood pressure. Later, a decline in serum catecholamine levels and peripheral vascular resistance is observed, which finally results into a cardiovascular collapse owing to hypovolemia.^7, 8^ In face of deteriorating hemodynamics, a compromised abdominal organ perfusion and reduced oxygenation is becoming evident.^9^ Accordingly, a shift from aerobic to anaerobic metabolism and acidosis is registered, clinically reflected by elevated serum levels of lactate and free fatty acids, and further promoted by decreased insulin secretion and hyperglycemia.^9, 10^ Activation of proinflammatory signaling pathways is finally observed.^9, 11, 12, 13, 14, 15^

On the other hand, it should be considered that both the magnitude of the catecholamine response and the extent of the myocardial injury seem to be correlated to the velocity of increase in ICP. Indeed, in experimental models of BD, an explosive increase in ICP resulted in increased plasma catecholamine, accompanied by extensive heart ischemic injury within 1 h after BD. However, a gradual increase in ICP, inducing BD after 2.5 h, resulted in lower increases in plasma catecholamine levels, and only mild heart ischemic injury.^16^ Consequently, from our point of view, further experimental studies are required to elucidate the relevance of the different magnitude of the catecholamine released consequently to BD on liver grafts undergoing transplantation. This would probably induce different inflammation and damage degree and differential underlying pathophysiological mechanisms in liver grafts undergoing transplantation. Thus, under these clinical situations, different protective treatments might be required to reduce the detrimental effects of BD in LT.

In the same line, different results related to the hormonal changes due to anterior and posterior pituitary failure have been described.^12, 17, 18, 19, 20, 21^ Indeed, it has been reported that anterior pituitary function seems to be well preserved in most donors with normal values of thyroid-stimulating hormone, adrenocorticotropic hormone and human growth hormone. Other authors described that, following BD, the function of the pituitary gland can remain active to a variable degree. However, other results indicate that when ICP exceeds the mean arterial pressure, brain perfusion stops, the pituitary gland is damaged and its hormone secretion ceases. Considering the aforementioned data, it would be of clinical interest to assess how the damage degree in pituitary gland may induce variability in the baseline values of plasma hormone and in the pattern of reacting hormones to overall reduce the deleterious effects of BD in LT. In addition, further experimental studies will be required to elucidate whether the variability in these hormonal changes predominantly depends on the length of time between the appearance of the noxious cause and surgery, or might be due to the etiology of BD.

## Strategies for Reducing BD Consequences in LT

In a prospective randomized trial with 100 BD donors, Kotsch *et al.*^22^ reported positive outcomes in LT after administration of steroids such as methylprednisolone owing to a downregulation in proinflammatory cytokines and reduced incidence of acute rejection. In contrast, another study in 90 BD patients failed to identify a benefit of methylprednisolone administration.^23^ Despite the heterogeneity of outcomes in clinical trials, steroid administration has been incorporated into donor management protocols,^24, 25, 26, 27^ and the regimen most frequently used for steroid treatment involves large doses of methylprednisolone. However, there is concern that high doses of steroids may worsen hyperglycemia, which may itself be detrimental to organ function^28^ (Table 1). In line with this, in an experimental study by Rebolledo *et al.*,^29^ the administration of prednisolone had different and disparent effects on liver allografts. Overall, there is a clear need to establish the effects of steroid treatment on liver grafts. Moreover, no conclusive data regarding this topic in marginal livers has been published.

Different results have been reported regarding the treatment with catecholamines. An experimental study indicated that the combined administration of epinephrine and vasopressin had a synergistic effect in improving the hemodynamics and maintenance of energy status of the liver. In a study based on 755 liver transplants performed in 26 hepatic transplantation centers, donor treatment with catecholamines (dopamine, epinefrine and norepinefrine) (separately or in combination) had little benefits on LT outcomes,^30^ whereas other paper including 12 LT reported adverse outcome following catecholamines treatment.^31^ The proinflammatory effects of norepinephrine increasing CXCL1 and IL-1 and the detrimental effects of dopamine impairing liver metabolism by reducing the redox state of liver mitochondria observed in experimental BD conditions^32, 33^ may explain the negative effects of catecholamines in LT (Table 1).

Different reports have also been published on the relevance of hormonal changes consequently to BD. For instance, thyroid hormone replacement therapy is a controversial part of donor management. In fact, experimental studies in BD organ donors by Novitzky *et al.*^10^ indicated that T3 treatment reduced lactate and free fatty acids in plasma, suggesting improvements in metabolic status.^34^ However, in a review by Powner and Hernandez,^35^ based on meta-analysis studies of more than 1000 patients, it was concluded that routine replacement of thyroid hormones for all donors could not be advocated (Table 1).

Given all these data into account, the strategies applied in LT have been mainly focused in the treatment with different hormones to stabilize the hemodynamic disorders associated with BD, whereas their effects on liver graft function and viability remain to be elucidated. In addition, different results have been found when comparing clinical studies, and comparing the few experimental studies reported with the clinical studies. Future experimental and clinical investigations will be required to optimize the management of BD organs to provide a hemodynamic stability during BD without adverse side effects for liver grafts and recipients. Moreover, it should be considered that a large number of factors and mediators also have a role in the mechanisms responsible for the detrimental effects of BD in LT; thus, strategies focused exclusively in providing hemodynamic stability during BD may be insufficient to prevent the deleterious effects of BD in LT.

## Marginal Livers from Brain Dead Donors for Transplantation

There are two categories of marginal livers,^36^ (A) livers that carry a high risk of technical complications and impaired function (i.e. elderly donors, steatotic donors or split livers) and (B) grafts that carry a risk of transmission of infection or malignancy to the recipient (i.e. donor with viral infections or donors with malignancy).

Despite numerous retrospective studies, the impact of each donor variables on graft function and recipient survival is still under investigation because of the contradictory results. Some investigators have indicated comparable results regarding graft function and patient survival after transplantation of marginal donors versus standard grafts, but most reports support a clear correlation between graft quality and posttransplant outcome.^37, 38^ Actually, the use and acceptance of marginal livers varies between different transplant centers^39^ and depends on the judgment of the transplant surgeon. Thus, retrieval teams may be cautious when accepting marginal organs for transplantation.

Multiple methods are currently being investigated to minimize the effects of ischemia–reperfusion (I/R) injury to allow the use of marginal organs, including anti-inflammatory approaches to attenuate cytokines, blockade of adhesion molecules, antiapoptotic strategies, among others. However, these studies are performed in non-BD surgical conditions and have been focused manly in steatotic or aged livers. Only a recent study, as described below, describes a potential treatment in both steatotic and non-steatotic liver grafts undergoing LT from cadaveric donors.

### Marginal livers with high risk of technical complications and impaired function

Donor age steadily increased over recent decades. In 1994, only 20% of deceased donors were 50 years or older. This percentage increased by >150% in the year 2004.^40^ Although initial studies suggested that donors >50 years old (without additional risk factors) have similar outcomes compared with younger donors,^41, 42^ and therefore age should not be a contraindication to liver donation, later reports concluded the contrary. Indeed, Busquets *et al.*^43^ reported that liver grafts from donors >70 years old had a higher risk for long-term graft failure and mortality, and more recent studies using large databases and different registries clearly identified donor age as an important risk factor related to graft failure and patient mortality.^44^

The few experimental studies of LT performed with old donors have been performed in non-BD surgical conditions. In such studies, it has been shown that livers from aged donors are more susceptible to endothelial cell injury and show impaired energy metabolism and reduced blood flow compared with younger livers.^45, 46^ Future studies will be required to evaluate the influence of these two factors, aging and BD, separately or in combination, in experimental models of LT, and therefore elucidate how BD may affect these liver grafts. Indeed, considering that the mechanisms responsible for I/R damage might be different (or more exacerbated) in older livers, different drugs or different drug doses should be administered in both young and old livers to protect them effectively against the detrimental effects of BD.

Regarding steatotic liver grafts, it has been reported that an estimated 20% of persons who accidentally die suffer from a mild or moderate grade of liver steatosis.^47, 48, 49^ Given the prevalence of hepatic steatosis in the society, this represents a large potential pool of donors. However, the clinical problem is still unresolved as steatotic livers are more susceptible to I/R injury and, when used, have poorer outcome than non-steatotic livers.^47, 50, 51, 52^ Indeed, LT outcome depends on the degree and type of hepatic steatosis;^53, 54^ however, in the transplant setting, a method for determining and measurement, the extent of steatosis remains imprecise and inconsistently reported,^55, 56^ and thus additional surrogate markers of organ quality are needed.

Experimental studies in steatotic livers have mainly focused on aggravated I/R injury after transplantation without considering BD, even though in clinical practice around 80% of grafts are taken from brain-dead donors. In such studies, lower antioxidant capacity, higher levels of cytokine release, Kupffer cell activation and leukocyte recruitment and a compromised microcirculation were observed in steatotic liver grafts undergoing transplantation compared with non-steatotic ones.^57, 58^ Only two studies in control animals, without transplantation, have evaluated the effects of BD in steatotic livers. In these studies, during BD-induced hypotension, portal venous and hepatic microvascular blood flow were reduced in steatotic livers compared with non-steatotic ones.^59^ In our opinion, as we will explain below, studies aimed at evaluating the pathophysiology of I/R associated with LT and establishing potential protective strategies in different types of livers should be performed in the presence of BD. Indeed, a recent experimental study from our group indicated that the injurious effects of BD were more exacerbated in the presence of moderate steatosis and occurred before liver grafts were retrieved from donors. In addition, the mechanisms responsible for the detrimental effects of BD were different depending of the type of the liver, which would interfere with protective pharmacological or surgical strategies applied in liver grafts, avoiding its potential benefits. In such a study,^60^ BD induced dysfunction in the cholinergic anti-inflammatory pathway and prevented the benefits induced by ischemic preconditioning (PC), a surgical strategy that shows benefits when it is applied in non-BD clinical situations such as hepatectomies. In fact, the study indicated that the combination of acetylcholine (ACH) and PC could be considered as a feasible and easy-to-perform intervention to reduce the adverse effects of BD and improve the quality of liver grafts. This specific strategy reduced the postoperative complications and increased the survival of recipients from steatotic and non-steatotic liver grafts from cadaveric donors. The advantages of combining a pharmacological and surgical strategy (ACH+PC), over a pharmacological strategy alone (ACH), might derive from the fact that PC involves a substantial number of molecular pathways that promote cellular resistance to stress, which is not observed when using a pharmacological treatment alone.^60^ Future studies are required to investigate whether the results obtained in an experimental model of esteatosis induced by obesity may also be extrapolated to other experimental models of liver esteatosis; as in clinical practice, the causes of hepatic steatosis are varied and include obesity, aging, moderate alcoholism, diabetes mellitus, hyperlipidemia and postmortem nutritional changes.

The use of split livers in transplant may be an option to expand the donor pool in cadaveric donors and it is possible in about 15% of optimal deceased donors.^61^ However, different clinical results have been reported when split livers are used for transplant.^62, 63, 64^ In a small series of split LT, 10 out of 12 adults receiving small grafts showed correct liver function posttransplantation.^62^ However, high rates of hepatic artery thrombosis, primary non-function and biliary complications, as well as problems associated with small-for-size syndrome, have been extensively reported.^64, 65, 66^ In our opinion, the detrimental effects of I/R injury on liver regeneration may be more exacerbated in the presence of BD. Our hypothesis is based on the following observations: split LT have a high risk of small-for-size syndrome,^64^ as the liver mass is not sufficient to meet the metabolic demands for the individual; in addition, I/R inherent to LT negatively affects regenerative responses and BD further detriments I/R process in LT. Evidently, future investigations on this topic aimed to establish protective strategies in this type of LT are still necessary.

### Marginal liver grafts with risk of transmission of infection or malignancy to the recipient

Whereas there are reasonable doubts about the use of split livers, grafts from old donors or with steatosis, there are still more questions to decide whether or not liver grafts form donors with viral infections or malignancies should be used for transplantation. In the specific scenario of hepatitis C virus (HCV) infection, and obviating the upcoming novel results using the newly developed therapeutic regimens, a relatively frequent retransmission of HCV to the recipient after LT, with concomitant morbidity and mortality, has been described.^67^ Similarly, the transmission of other donor-derived malignancies with detrimental outcomes has also been reported.^68, 69, 70^ Thus, it is left to the judgment of the transplanting team to determine the use of these organs under certain circumstances.

Up to now, one of the most controversial issues regarding extended-criteria donors resolves around the potential positive impact of HCV-infected donors on short-term outcomes. Donor seropositivity for HCV has been considered a contraindication for LT and not commonly practiced. However, it has been reported that 1-year patient survival rates of 97% in recipients of HCV-infected livers compared with rates of 87% for recipients of organs meeting the United Network for Organ Sharing-approved criteria, with no differences in surgical conditions including warm and cold ischemia times between both groups.^71^ If the results of this study are validated by others, it might have an important clinical implication because such organs are underused but overpresented in the donor pool. Again, the new panorama after the administration of the new generation of anti-HCV therapies might very much change the future in this field, requiring subsequent analysis and characterizations.

## Future Perspectives and Conclusions

**Figure 3:**
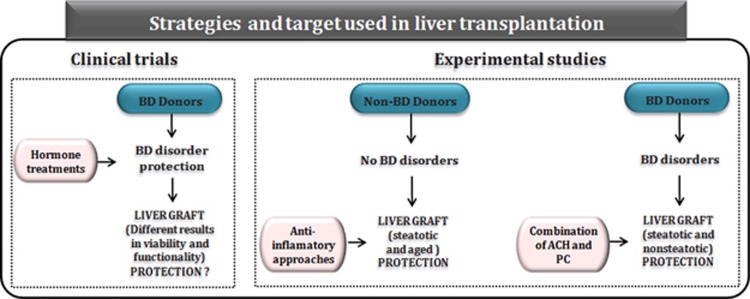
Therapeutic strategies evaluated in clinical and experimental models of LT. Bedside (left), different hormone treatments targeting hemodynamic deterioration owing to BD have shown diverse results. Benchside (right), majority of the experimental studies evaluated therapeutic strategies to protect livers undergoing I/R injury without the presence of BD. Contrarily, a strategy based on the combination of ACH and PC revealed marked protection in the presence of BD

Most organs for transplantation originate from brain-dead donors. Although the detrimental consequences of BD have been clinically described, the underlying mechanisms and their relevance in LT remain poorly understood. Indeed, few studies have evaluated the effect of BD on LT, and most of the experimental studies focused in establishing surgical or pharmacological strategies to reduce liver graft damage associated with transplantation have been performed in the absence of BD. Moreover, as stated along this review, different results on treatments, mainly focused on hemodynamic stabilization, have also been reported. On the other hand, owing to the persistent shortage of liver grafts for transplant, the use of marginal livers has been increased in the past years. However, clinical studies in LT are mainly descriptive and dissimilar results regarding the postoperative outcomes of LT when marginal livers are used have been reported. Multiple methods, in non-BD conditions, are currently being investigated to minimize the effects of I/R injury to allow the use of marginal livers for transplant. However, given the result from our recent study in steatotic and non-steatotic LT from cadaveric and non-cadaveric donors, we believe that the experimental conditions should maximally mimic the clinical situation of LT to develop ultimately effective therapeutic strategies in this setting. Such investigations should be focused not only on liver graft damage associated with transplantation but also on brain-dead donor, which may very much contribute to this pathology (Figure 3).

Future prospective, randomized clinical studies and studies that include a sufficient number of marginal donors will be required to elucidate the effect of BD on marginal liver grafts and to select the most appropriate marginal organs for transplant. Future research in experimental models of LT using BD donors is required to understand the pathophysiology of BD and elucidate the consequences of BD. These new studies should analyze the type and extent of brain injury using different times of cold ischemia and include the subanalysis of the impact of possible diseases present in the liver, with the ultimate goal to define novel and effective treatment targets. We recognize that a main difficulty when using marginal livers is to define the criteria that can be extended, because these criteria vary between centers and regions, but it is clear that different postoperative outcomes exist when marginal livers are used. Thus, further experimental research is also needed to identify better tests for evaluating the quality of donor organs. Obviously, all of this requires necessary additional efforts of multidisciplinary research groups.

## Figures and Tables

**Table 1:** Pharmacological and surgical strategies applied in liver transplantation from BD donors

**Study**	**Donors**	**Treatment**	**Dose**	**Results**
*Human studies*				
Kotsch *et al.*^22^	BD patients	Methylprednisolone	250 mg Bolus and 100 mg/h infusion	↓ Proinflammatory cytokines and incidence of acute rejection
Amatschek *et al.*^23^	BD patients	Methylprednisolone	1000 mg Bolus	Not ameliorate liver allograft function, mortality or rejection
Schnuelle *et al.*^30^	BD patients	Catecholamines (separately or in combination)	Not specified	Little benefit is conferred after transplantation
Yamaoka *et al.*^31^	BD patients	Catecholamines	Not specified	↓ Ketone body ratio ↑ Detrimental effect on liver metabolism
Power *et al.*^35^	BD patients	Thyroid hormone replacement	0.2–2 *μ*g/kg Bolus or 0.05–2 *μ*g/kg per h infusion (T3) 20 *μ*g Bolus, not specified or uncontrolled (T4)	Not conclusive results on thyroid hormone replacement
				
*Animal studies*				
Rebolledo *et al.*^29^	BD rats	Prednisolone	22.5 mg/kg Bolus	↓ TNF-*α* and HSP70 expression Not reduced liver injury
Okamoto *et al.*^33^	BD dogs	Dopamine	15 *μ*g/kg per min infusion or more	↓ The redox state of liver mitocondria ↑ Detrimental effects impairing liver metabolism
Lewis *et al.*^32^	BD rats	Norepinephrine and vasopressin	82 *μ*g/kg per h and 0.27 U/kg per h infusion (norepinephrine and vasopressin, respectively)	↑ CXCL1 and IL-1*β* expression
Jimenez-Castro *et al.*^60^	BD rats	Combination of ACH and PC	500 *μ*g/kg Bolus (ACH)	↓ Adverse effects of BD and postoperative complications ↑ Quality of liver grafts and recipient survival

Abbreviations: ACH, acetylcholine; BD, brain death; CXCL1, chemokine (C–X–C) ligand 1; IL-1*β*, interleukin 1*β*; PC, ischemic preconditioning; TNF-*α*, tumor necrosis factor-*α*; HSP70, heat-shock protein 70

## Abbreviations

ACH: acetylcholine
BD: brain death
DBD: donation after brain death
DCD: donation after circulatory death
HCV: hepatitis C virus
ICP: intracranial pressure
PC: ischemic preconditioning
LT: liver transplantation
